# Evaluation of extension of breast screening to women aged 65–70 in England using screening performance measures

**DOI:** 10.1038/sj.bjc.6604981

**Published:** 2009-03-10

**Authors:** R L Bennett, R G Blanks, S M Moss

**Affiliations:** 1 Cancer Screening Evaluation Unit, Institute of Cancer Research, 15 Cotswold Road, Sutton, Surrey SM2 5NG, UK

**Keywords:** breast cancer, screening, older women

## Abstract

The objective of this study was to investigate screening performance measures in the English screening units that began inviting women aged 65–70 between 1 April 2001 and 1 April 2004. We analysed results after each unit commenced inviting women aged 65–70. In addition, we analysed data from units that invited this age group for a second time between 1 April 2004 and 31 March 2007. Results for women aged 65–70 were compared to women aged 50–64 and 60–64. Average uptake was 72.8% for women aged 65–70 and 76.7% for women aged 50–64. For women screened within the last 5 years, uptake was 88.7% for older women and 89.1% for younger women. For women previously screened within 5 years the invasive cancer detection rate was 17% higher in the 65–70 age group than in the 60–64 age group. The rates of recall to assessment and PPV were 3.5 and 27.6% in women aged 65–70 and 3.4 and 24.6% in women aged 50–64 respectively. These results suggest that, as in the earlier demonstration studies, uptake rates remain high in older women, and many more older women attend following an invitation than had previously self-referred. The cancer detection rate is higher in this older age group, whereas rates of recall are generally similar to those in younger women; consequently the PPV is also higher in older women.

When the UK NHS Breast Screening Programme (NHSBSP) began in 1988, women aged between 50 and 64 years were invited to three yearly screening, whereas older women were able to self-refer. (Forrest, 1986). A survey in 1995 showed that in many countries women aged up to 69 or 70 are invited to screening (Shapiro *et al*, 1998). Although breast screening is acknowledged as efficacious for women aged 50–69, when the UK programme commenced there was concern regarding possible lower uptake and reduced cost-effectiveness in older women (Moss *et al*, 2001). More recently, results from three demonstration studies in England, in which invitations were extended to women aged 65–69, suggested that older women who have previously been routinely screened by the programme had similar uptake patterns to younger women, and screening these women was estimated to be as cost-effective as for the 50–64 age group (Moss *et al*, 2001). As a result of these studies, the expansion of the programme to invite women up to the age of 70 was announced in the NHS Cancer Plan in 2000 (Department of Health, 2000). The publication also announced the introduction of two-view mammography at the incident screen. English units were expected to have begun this by 2003 and to have started including women aged 65–70 into the routine invitation system by the end of 2004.

The aim of this study was to investigate if the implementation of this policy change gave similar results to those from the demonstration studies. We analysed results from those units that began inviting the 65–70 age group between 1 April 2001 and 1 April 2004 and therefore had completed at least a full 3-year screening round by 31 March 2007.

## METHODS

Between April 2001 and April 2004, 36 units in England began inviting women aged between 65 and 70, in addition to women aged 50–64, to screening. These units were distributed throughout England and represented approximately 40% of both the total number of units and of the target population of the screening programme.

Aggregated information on activity and outcomes is reported annually by screening units on statutory KC62 returns, which report on specific cohorts of women; for example data for 2002 relate to women screened between 1 April 2001 and 31 March 2002.

As the majority of women aged 65–70 will have been previously screened, performance measures (rates of recall, cancer detection, benign biopsy and non-operative diagnosis of cancer) were calculated for women who had previously been screened by the programme both within the last 5 years and more than 5 years previously. Uptake of invitation was calculated for all women according to previous screening history. Results for the 65–70 age group were compared with those for both the previous target age range (50–64) and the oldest age group in that range (60–64). The statistical significance of these comparisons was tested using a two-sample test of proportions in Stata version 9.2.

We examined data for each unit for the first 3 complete years after they commenced inviting the 50–70 age group. For example, in a unit beginning to invite the 65–70 age group in August 2003, we used data for the period 1 April 2004 to 31 March 2007. To estimate the increase in the number of older women being screened due to the expansion of invitation to the 65–70 age group, we compared the number of women aged 65–70 screened in this 3-year period with the number of women who self-referred in the 65–69 age group (because data for age 70 were not available before 2003) in the 3 complete years prior to each unit inviting older women.

**Figure 1:**
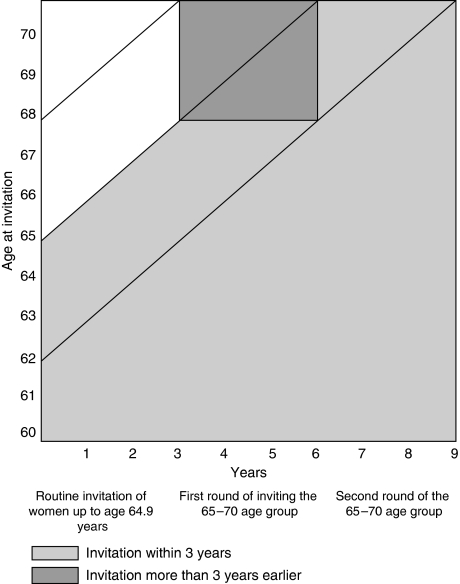
Age at invitation and previous screening history of older women in units commencing invitation of 65–70 age group.

During initial invitation of older women (round 1), those aged between 65 and 67 will have been invited to screening 3 years previously, and are likely to form the majority of women screened within the last 5 years, whereas those aged 68–70 are likely not to have been invited for 6 years and will form the majority of women last screened more than 5 years previously. In addition, for the 23 units that began the expansion between April 2001 and April 2003, we examined data as they began the re-invitation of the 65–70 age group (round 2) to screening, when all women will have been invited to screening within the last 3 years. Figure 1 shows the previous screening history of women aged 65–70 at both rounds 1 and 2 assuming that before the policy change women had received their last invitation between ages 62 and 64.9 years.

In 2004, an annex was added to the data return providing anonymised individual-based pathology data for all screen-detected cancers. The quality of these data was poor in the first year, and we therefore used data from 2005 onwards. Data on all cancers detected by the screening units during the 3-year period between 1 April 2004 and 31 March 2007 (KC62 year, 2005–2007) were used to calculate the Nottingham Prognostic Index (NPI) in order to compare the prognosis for the 65–70 age group with that of younger women. The index was derived from a series of women with primary breast cancer and uses lymph node status, tumour size and pathological grade. From this series three subsets of patients were identified; those with a good prognosis, a moderate prognosis and a poor prognosis (survival at 10 years after diagnosis was 88, 60 and 18% respectively) (Blamey, 2002).

## RESULTS

**Figure 2:**
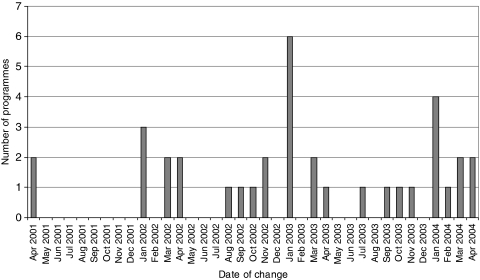
Start date for programmes commencing to invite 65–70 age group between April 2001 and April 2004.

The dates when the 36 units began inviting women up to the age of 70 are shown in Figure 2. Units commencing between April 2001 and April 2003 began re-inviting the 65–70 age group between April 2004 and April 2006. Although two units commenced inviting the 65–70 age group in April 2001, the majority of the units began in 2003. By this time approximately 60% of the units had already started taking two views at the incident screen, and a further third started at the same time; therefore most women invited by the units would have had two mammographic views taken.

A total of 516 213 women aged 65–70 were invited to screening in the first round, and 201 589 in the second round. Uptake for this age group was 72.8% at both rounds. Average uptake in the two rounds was 76.7%, and 76.8% for the 50–64 and 60–64 age groups respectively. The number of older women screened in the first round was more than five times the number (67 546, aged 65–69) who self-referred in these units in the 3 years prior to them beginning to invite older women.

Table 1 shows the uptake rates by age group, invitation type and round. A percentage of women aged 65–70 were invited for the first time. (Reasons for this may include women moving to the UK, previously screened women moving to a new area whose screening history was not forwarded from their old area and women previously recorded as ineligible now being invited). At the first round, the 65–70 age group demonstrated similar patterns of uptake to women aged 60–64, except for those women whose last screen was more than 5 years ago, whose uptake was higher (66.6 *vs* 46.0%). The results may reflect the fact that the older women in the 65–70 age group would not have been invited for 6 years. A proportion of older women in this age group will have been screened more recently as a result of self-referral, and this may explain why more than half of the 65–70 age group had been screened within the last 5 years. At the second round, for women screened more than 5 years previously, the older age group showed a similar pattern of uptake to the 60–64 age group; all these women will have been non-attenders at their last invitation.

The overall recall to assessment rate in the 65–70 age group was 3.4% for women screened within the last 5 years and was similar to the 60–64 age group at both rounds (Table 2). For women screened more than 5 years previously the rate was 5.2% in the older age group. This was similar to rates in the younger age groups at the first round but at the second round rates decreased in both the 50–64 and 60–64 age groups but remained higher in the 65–70 age group.

The average invasive cancer detection rate in the 65–70 age group was 7.6 per 1000 in women screened within the last 5 years and 12.0 per 1000 in women screened at a longer screening interval (Table 2). Both were significantly (*P*<0.001) higher than rates in the 60–64 age group (6.5 per 1000 women screened and 8.9 per 1000 women screened respectively).

The *in situ* cancer detection rate was similar in the 60–64 and 65–70 age groups; 1.6 per 1000 women screened *versus* 1.7 per 1000 women screened for women screened within 5 years and 2.2 per 1000 women screened *versus* 2.6 per 1000 women screened in women screened more than 5 years ago (Table 2).

In women screened within the last 5 years the small (<15 mm) invasive cancer detection rate was significantly (*P*<0.001) higher in the 65–70 age group than the 60–64 age group (4.4 per 1000 women screened *vs* 3.7 per 1000 women screened) (Table 2). Rates were also higher in older women screened more than 5 years ago but the rates decreased from the first to the second round in all age groups (unlike in women screened within the last 5 years where they remained stable).

The percentage of small (<15 mm) invasive cancers was 55% in the 50–64 age group, 57% in the 60–64 age group and 58% in the 65–70 age group for women screened within the last 5 years. For women screened more than 5 years previously the percentages were 53, 52 and 51% respectively.

The positive predictive value of recall to assessment (PPV) was significantly (*P*<0.001) higher in the 65–70 age group than the 60–64 age group for women screened both within the last 5 years (27.6 *vs* 24.6%) and those with a longer screening interval (28.3 *vs* 21.8%).

Rates of benign surgical biopsies for the 65–70 age group were 0.06% in women screened within 5 years and 0.10% for women screened more than 5 years previously. These were similar to those in the 50–64 and 60–64 age groups. The overall percentages of cancers with a non-operative diagnosis of cancer were also similar between the 60–64 and 65–70 age groups with 95.1% of older women screened within 5 years receiving a non-operative diagnosis of cancer and 94.8% who were screened more than 5 year previously.

Table 3 shows the proportion of cancers (including those with an unknown lymph node status) falling into each of the diagnostic categories of the NPI. The proportion of cancers in older women with a good prognosis was similar to that in the 60–64 age group.

## DISCUSSION

Although units included in this study began including women aged 65–70 in the invitation system between April 2001 and April 2004, most began inviting older women after December 2002; results from the first invitation of the 65–70 age group therefore mainly represented activity between 1 April 2003 and 31 March 2007, whereas the results for the re-invitation of the 65–70 age group were based on units that began earlier and represent activity mainly between 1 April 2005 and 31 March 2007. The re-invitation of the age group only represents a full 3-year screening round for 2 out of the 23 units. However, average performance measures in England between April 2003 and March 2007 were stable apart from recall rate which has decreased.

The results of this study showed that although uptake was lower in women aged 65–70 than in women aged both 50–64 and 60–64 for all age groups, it was more than the 70% minimum target set by the programme (NHS Breast Screening Programme, 2005). The results are similar to those from the English demonstration studies and results from screening programmes in both Denmark and the Netherlands, which also invite women aged 65–69 and report more than 70% of women accepting an invitation to screening (Sarkeala *et al*, 2004).

The invasive cancer detection rate in women screened both within 5 years and more than 5 years previously and the small invasive cancer detection rate in women screened more than 5 years ago both decreased between the first and second rounds. At the first round, in the 50–64 age group, these rates were higher than the English average and thus results at the second round, although based on fewer data, may better reflect rates as units continue to invite women aged 65–70.

As in the demonstration studies, the rise in breast cancer incidence with age was reflected in the increased invasive cancer detection rate in the 65–70 age group. However rates of both small and overall invasive cancers in all age groups were higher in this study than in the demonstration studies, probably as a result of units now using two-view mammography at all incident screens. However, a slower rate of tumour growth is suggested by an increased mean sojourn time in the older age group (Tabar *et al*, 2000), this may suggest an increasing chance of overdiagnosis in the older age group due to increasing all-cause mortality.

As a result of the increased invasive cancer detection rate in the 65–70 age group, and low rates of recall to assessment, the PPV increased in older women. However, results from further units will be watched with interest. The demonstration studies showed a lower rate of recall in the older age group, although the Netherlands, which has always invited the 50–70 age group, has shown an increase in recall rate, as well as PPV and cancer detection, with increasing age (National Evaluation Team for Breast Cancer Screening, 2005).

In 2002, an IARC working group concluded, given the evidence from randomised controlled trials, that breast screening programmes should target women aged 50–69 (IARC Working Group on the Evaluation of Cancer-Preventive Strategies, 2002). Some countries now routinely invite women aged over 70; women are invited up to 74 years in Sweden and 75 years in the Netherlands. In England, the recent publication of the Cancer Reform Strategy announced the further expansion of the NHSBSP to women aged 47–73 years (Department of Health, 2007). The results from this observational study of extending the upper age limit to 70 have shown that although women above the age of invitation are able to self-refer, the number doing so is far fewer than the number of women in the same age group who attend following an invitation. Consequently, current rates of self-referral in women aged 71–73 should not be used to predict future uptake in this age group. The effect on resources of the further extension of the programme is likely to be considerable if uptake for both the 71–73 and 47–49 age groups is similar to the 50–70 age group.

### Calculation of the NPI

The index was calculated using the formula: size (cm) × 0.2+lymph node status (1–3)+grade (Haybittle *et al*, 1982). An index using a revised formula (size (cm) × 0.42+grade × 0.78) was calculated for cancers with unknown lymph node status (The Breast Screening Frequency Trial Group, 2002).

## Figures and Tables

**Table 1:** Uptake of screening according to invitation type and screening round

	**First round of inviting 65–70 age group**		**Second round of inviting 65–70 age group**		**Overall**	
**Invitation type**	**Invited**	**Uptake (%)**	**Invited**	**Uptake (%)**	**Invited**	**Uptake (%)**
*First invitation*						
50–64	360 432	74.0	130 075	73.0	490 507	73.7
60–64	7836	35.3	2713	36.8	10 549	35.7
65–70	8209	31.7	2269	30.1	10 478	31.4
						
*Previously invited never screened*						
50–64	194 771	21.0	77 044	19.7	271 815	20.6
60–64	50 689	10.1	20 590	9.6	71 279	9.9
65–70	50 349	8.5	18 599	6.6	68 948	8.0
						
*Previous screen ⩽5 years*						
50–64	1 244 889	89.3	471 812	88.6	1 716 701	89.1
60–64	434 364	89.8	175 411	89.5	609 775	89.7
65–70	288 718	88.9	147 893	88.3	436 611	88.7
						
*Previous screen >5 years*						
50–64	128 460	51.5	50 399	49.9	178 859	51.1
60–64	61 404	46.0	25 054	44.9	86 458	45.7
65–70	168 937	66.6	32 828	43.7	201 765	62.8

**Table 2:** Rates of recall to assessment and cancer detection by screening round, previous screening history and age group

			**Cancer detection rate per 1000 women screened**		
	**No of women screened**	**Recalled to assessment (%)**	**Invasive cancers**	***In situ* cancers**	**Small (<15 mm) cancers**
*First round of inviting 65–70 age group*					
Women previously screened ⩽5 years ago					
50–64	1 111 069	3.4	5.3	1.4	2.9
60–64	390 198	3.4	6.6	1.6	3.7
65–70	256 671	3.5	7.8	1.7	4.4
Women previously screened >5 years ago					
50–64	66 172	5.2	8.1	2.1	4.4
60–64	28 220	5.4	9.0	2.3	5.0
65–70	112 432	5.2	12.0	2.6	6.2
					
*Second round of inviting 65–70 age group*					
Women previously screened ⩽5 years ago					
50–64	417 983	3.1	5.1	1.4	2.9
60–64	156 971	3.1	6.2	1.5	3.6
65–70	130 594	3.1	7.2	1.6	4.4
Women previously screened >5 years ago					
50–64	25 146	4.8	7.7	2.2	3.8
60–64	11 255	4.5	8.5	2.0	3.9
65–70	14 339	5.3	11.6	2.4	5.4

**Table 3:** NPI (prevalent and incident screens combined) by age group

	**Good**		**Moderate**		**Poor**		**Not known**		
	**No**	**%**	**No**	**%**	**No**	**%**	**No**	**%**	**Total**
50–54	1313	55	891	37	136	6	65	3	2405
55–59	1503	53	1058	37	192	7	89	3	2842
60–64	1715	58	1007	34	147	5	112	4	2981
65–70	2023	59	1110	32	165	5	128	4	3426
